# Supplementary material for: Learning curve and surgical outcome of robotic assisted colorectal surgery with ERAS program
Source: Sci Rep. 2022 Nov 29;12:20566. doi: 10.1038/s41598-022-24665-w (PMC9709162; doi:10.1038/s41598-022-24665-w)
Supplement: Supplementary file 2 — Supplementary Information 2. [file 41598_2022_24665_MOESM2_ESM.zip › RAL Raw data-Ñ[▒K/088/HS_ñw╝╨░O▒Kñσ.pdf]

正式摘要(電子簽章：000000218639634)

W95 / W95 ☐ 英文標題(注意:無英文姓名-請補病人基本檔!) ☐【讀取】憑證

臺中榮民總醫院

出院病歷摘要

轉入號： 轉出號：

[redacted]

(8)流水編號	
(9)入院日期	110/06/01 (2021) 直腸外科 W95 - 33
(10)轉科日期	
(11)出院日期	110/06/06 (2021) 住院天數計 5 日

診斷

- 入院 :A-colon cancer 4.5cm, with regional nodal metastasis
  [Hx]
  1. HTN
  2. Gout without medication
  3. Hemorrhoid, s/p internal ligation
- 出院 :A-colon adenocarcinoma 5x4 cm, with mesocolic lymph nodes metastasis, cT4aN2aM0
  s/p Robot assisted right hemicolectomy + D3 dissection on 2021/06/02.
  pT3N2a(6/39, loca5/25, RC 1/2, SMV 0/5)M0, pMMR, MD, PI+, AI-, TuDp-, CRM-1.5cm, ExNd+. stage IIIB
  [Hx]
  1. HTN
  2. Gout without medication
  3. Hemorrhoid, s/p internal ligation

主訴

- anal pain with occasional anal bleeding for two months

病史

- The 61-year-old male had history of
  1. HTN
  2. Gout without medication
  3. Hemorrhoid, s/p internal ligation

  According to the patient, he suffered from anal pain with occasional anal bleeding for two months. He went to Kuang Tien H. for help, where hemorrhoid was found, status post internal ligation. However, the symptoms persisted. Therefore, he came to LMD for second opinion, where colonscope was done, showing A colon tumor, s/p biopsy on 2021/05/28. Tumor marker was under normal range, told by LMD doctor. The pathological report is pending. Due to the patient request, he was transferred to our OPD.
  This time, under the impression of A colon tumor, the patient was admitted for robotic-assisted colectomy.

體檢發現

-
  - General appearance :
    - easy looking , conscious clear , without cardiopulmonary distress
    - Vital signs自動帶入date 體溫(BT) 脈搏(P) 呼吸(R) 血壓(BP) 身高(BH) 體重(BW) BMI
      20210601 36.6℃ 100次/分 18次/分 115/84mmHg 159.9cm 66.7kg 26.1
    - Vital signs: B.P. 115/84 mmHg , P.R. 100 /min , R.R. 18 /min , B.T. 36.6 ℃
  - HEENT :
    - Eyes: no anemic conjunctiva, no icteric sclera, normal EOM
    - ENT: no otorrhea, no rhinorrhea, no erythematous mucosa, no injected tonsil, no deformity
  - Neck: supple, freely movable, trachea midline,no deformity, no engorged jugular vein , no palpable thyroid or lymph node enlargement
  - Chest:
    - Inspection: Grossly normal, symmetric and fully expansion, no accessory muscle used, no operation scar, no spider angiomata, no gynecomastia
    - Palpation: no palpable mass or nodule, no subcutaneous emphysema, no tenderness
    - Ausculation: clear breathing sound
  - Heart:
    - Percussion: normal heart size
    - Ausculation: regular heart beat, normal S1, S2, no gallop, no murmur
  - Abdomen:
    - Inspection: in
      - flat contour, symetrically, no scar, no stria, no dilated veins, no rashes
    - Ausculation: normoactive bowel sound, no abdominal bruits
    - palpation: soft, no muscle guarding or rigidity, no local tenderness, no rebounding pain, no palpable mass, no palpable pulsation
    - Percussion: no shifting dulllness, no hepatomegaly, no splenomagaly
  -

Peripheral pulsation :	CA	BA	RA	FA	PA	PTA	DPA
Right side :	++	++	++	++	++	++	++/+++
Left side :	++	++	++	++	++	++	++/+++

  - Genito-anus: no tenderness or palpable mass lesion or rectal shelf, normal external genitalia, normal anal tone, brown stool

CRS CONDITION

  -
    - Lung

手術日期及方法

- 手術日期及方法20210602 Robot Assisted Colectomy(R)

住院治療經過

- Scheduled operation of robotic-assisted colectomy was done smoothly on 6/2, and the patient could tolerate it well. He began to try warmI diet on 6/4, warmII diet on 6/5. Under relatively stable condition, he was discharged with OPD follow up.

合併症

一般檢查

- 累積報告 :

DATE	TIME	ALB	TP	BIL.T	BIL.D	ALKP	AST	ALT	LDH	AMY	LIPASE	NH3	Ferritin
1100603	0847			0.70	0.19		14	8					

DATE	TIME	WBC	RBC	HGB	HCT	MCV	PLT	BLST	NEUT	BAND	SEG	LYM	MON	EOS	BAS
1100603	0917	14390	3.56	11.2	32.8	92.1	317		73.6			18.9	7.2	0.1	0.2

DATE	TIME	NA	K	CL	BUN	CREAT	eGFR	CA	Mg	P	CRP	ESR	PCT	D-Dimer	LACTATE
1100603	0847	139	3.8		13	1.39		8.2							

- 放射線報告 :檢驗項目 **:CT ABDOMEN-SURVEY** 報告日期 **:1100601**
  主 訴：
  Result:Y
  Finding:
  Imaging Report Form for Colorectal Carcinoma (AJCC 8th)
  1. Imaging Date: 20210531
  Imaging Modality, Imaging by
  CT scan

- Imaging Protocol: CT scan of abdomen with IV and oral contrast enhancement:
 2. Tumor location / Size
 Location:
 Ascending
 Size:
 Measurable: 4.5 cm (largest diameter)
 3. Tumor invasion
 Tumor invades visceral peritoneum or adjacent organs
 4. Regional nodal metastasis
 N2 | N2a Four to six regional lymph nodes are positive.
 5. Distant metastasis (In this study)
 No or Equivocal
 6. Other findings: > A case of r/o colon tumor
 > Poor enhancing lesion about 1.5 cm is noted over left adrenal glands, suggest further evaluation
 > A metallic clip noted within A colon with irregular wall thickening and some small lymph nodes noted over abutting mesocolon. Colon cancer is suspected. Suggest correlate with colonsocopy.
 > Air density is noted of left aspect of L5/S1 IVD
 > No definite focal lesion in liver parenchyma, spleen, pancreas, gallbladder, and kidneys.
 > No significant enlarged lymph nodes over retroperitoneal region.
 > The lower abdomen showed no significant unusual.
 Impression:
 A metallic clip noted within A colon with irregular wall thickening and some small lymph nodes noted over abutting mesocolon. Colon cancer is suspected.

 檢驗項目 **:ABDOMEN (KUB),SUPINE** 報告日期 **:1100601**
 主 訴：
 Result:N
 Finding:
 > Some fecal material and bowel gas retention within colon.
 > Bilateral psoas shadows are clear in appearance.
 > DJD along lumbar spine with spur formation.
 > Surgical clips over right upper abdomen, may due to previous surgical history.
 Impression:
 As above description.

 檢驗項目 **:CHEST PA VIEW** 報告日期 **:1100531**
 主 訴：
 Result:N
 Finding:
 1. The heart size is within normal limit.
 2. Tortuosity and atherosclerotic changes of T-aorta.
 3. No definite active lung lesions.
 4. DJD changes with marginal spur formation of T-L spine.
 Impression:
 As above description.

特殊檢查

病理報告

- 活體組織切片病理報告　手術日期：**110/06/02**　病理號：**S1018199**

 診斷醫師:陳韻安 報告日期:110/06/09
 Pathologic diagnosis:
 1. Colon, terminal ileum, and appendix, right hemicolectomy --- Adenocarcinoma, moderately differentiated, of ascending colon. Tumor invaded to mesocolic soft tissue and metastasized to mesocolic lymph nodes (5/25). The appendix, ileocecal valve and bilateral cut ends are free of tumor.
 2. Lymph node, IC, dissection --- Reactive lymphoid hyperplasia (0/7).
 3. Lymph node, right colic, dissection --- Metastatic adenocarcinoma (1/2).
 4. Lymph node, SMV, dissection --- Reactive lymphoid hyperplasia (0/5).
 Ancillary study for diagnosis:
 Deep cut and IHC stains for PMS2 and MSH6 are done on section F. All above markers are preserved.
 Prognostic and predictive factor:
 1. Tumor size: 5x4 cm.
 2. Depth of invasion: Pericolorectal tissue.
 3. Histologic grade: G2: Moderately differentiated.
 4. Angiolymphatic invasion: Not identified.
 5. Perineural invasion: Present.
 6. Tumor deposits (discontinuous extramural extension): Not identified.
 7. Macroscopic tumor perforation: Not identified.
 8. Surgical Margins: a) Proximal Margin: Uninvolved by invasive carcinoma, distance of tumor from margin: 11 cm. b) Distal Margin: Uninvolved by invasive carcinoma, distance of tumor from margin: 3 cm. c) Radial or Mesenteric Margin: Uninvolved by invasive carcinoma, distance of tumor from margin: 1.5 cm.
 9. Lymph node status: Involved (6/39).
 10. Extranodal involvement: Present.
 11. Tumor regression grading S/P CCRT: No known presurgical therapy.
 12. Type of polyp in which invasive carcinoma arose: Not identified.
 13. Additional pathologic findings: Not identified.
 14. Pathological TNM Stage: pT3N2a(According to the eighth edition, American Joint Committee on Cancer Staging Guidelines for Tumors).
 15. TNM descriptors: Not applicable.
 Gross description:
 The specimen consists of 1) a segment of colon, 13 cm in length, and 6.5 cm in proximal circumference, 6 cm in distal cut end circumference, and a segment of terminal ileum, 4 cm in length, and 3.5 cm in cut end circumference. An unremarkable appendix, 8 cm, is noted. A gray tan firm annular elevated and centrally ulcerative tumor, 5x4 cm, is noted, 3 cm to the distal cut end. On cross section, the tumor invaded to the mesocolon with 15 mm to the radial margin grossly, and is close to the serosa with serosal retraction. The appendix, ileocecal valve, and both cut ends are free of tumor. There is also a clip at the distal part of the ascending colon. No lesion can be found grossly around this clip. 2) a piece of yellow soft tissue, 7x4.2x1.3 cm, labeled as "IC lymph node". 3) a piece of yellow soft tissue, 6x3x0.5 cm, labeled as "right colic lymph node". 4) a piece of yellow soft tissue, 4x1.3x0.6 cm, labeled as "SMV lymph node". Representative part for section: A) proximal cut end B) distal cut end C) ileocecal valve D-E) appendix F-K) tumor (with G-H mapping) L) clip-labeled area M,N,P) sp.2,3,4.
 Note:
 1. The interpretation of MMR panel and recommended studies are as below: (1) No loss of nuclear expression of MMR proteins: low probability of microsatellite instability-high (MSI-H) . (2) Loss of

- nuclear expression of MLH1 and PMS2: testing for methylation of the MLH1 promoter and/or mutation of BRAF is indicated (the presence of a BRAF V600E mutation and/or MLH1 methylation suggests that the tumor is sporadic and germline evaluation is probably not indicated; absence of both MLH1 methylation and of BRAF V600E mutation suggests the possibility of Lynch syndrome, and sequencing and/or large deletion/duplication testing of germline MLH1 may be indicated). (3) Loss of nuclear expression of MSH2 and MSH6: high probability of Lynch syndrome (sequencing and/or large deletion/duplication testing of germline MSH2 may be indicated, and, if negative, sequencing and/or large deletion/duplication testing of germline MSH6 may be indicated). (4) Loss of nuclear expression of MSH6 only: high probability of Lynch syndrome (sequencing and/or large deletion/duplication testing of germline MSH6 may be indicated). (5) Loss of nuclear expression of PMS2 only: high probability of Lynch syndrome (sequencing and/or large deletion/duplication testing of germline PMS2 may be indicated). (6) Please note: There are exceptions to the above IHC interpretations. These results should not be considered in isolation, and clinical correlation with genetic counseling is recommended to assess the need for germline testing.
  2. This case has been peer reviewed by two doctors.
  #T-59420_2 #M-81403_2 2 1105 S0000F
  #T-C4560_2 #M-81406_2 2 1105 00000F
  #T-59200_2 #M-09183_2 0 1105 000000

其它

出院時情況

- 3.改門診治療

出院指示

藥名	劑量	劑量單位	用法	頻次	數量	數量單位	現況	天數	單位
(口)Takepron OD tab 30mg	1	TAB	PO	QDAC	10	TAB	TH	10	天
Voren-K tab 25mg	1	TAB	PO	TID	30	TAB	TH	10	天
Ultracet tab	1	TAB	PO	Q12H	20	TAB	TH	10	天

出院計劃

主治醫師 2222H 林俊余 / 住院醫師 2343L 張嘉育

正式摘要(電子簽章：**000000218639634)**

W95 / W95

doctorRank:B
